# Cinnamon extract improves abnormalities in glucose tolerance by decreasing Acyl-CoA synthetase long-chain family 1 expression in adipocytes

**DOI:** 10.1038/s41598-022-13421-9

**Published:** 2022-07-22

**Authors:** Tsubame Nishikai-Shen, Tomomi Hosono-Fukao, Toyohiko Ariga, Takashi Hosono, Taiichiro Seki

**Affiliations:** 1 Department of Chemistry and Life Science, College of Bioresource Sciences, Nihon University, Kanagawa, 252-0880 Japan; 2 Department of Applied Life Sciences, Nihon University Graduate School of Bioresource Sciences, Kanagawa, 252-0880 Japan; 3 Intractable Disease Research Center, Juntendo University School of Medicine, Tokyo, 113-8421 Japan; 4 Division of Regenerative Therapy, Juntendo University Graduate School of Medicine, Tokyo, 113-8421 Japan

**Keywords:** Biomarkers, Molecular medicine

## Abstract

We previously demonstrated that cinnamon extract (CE) alleviates streptozotocin-induced type 1 diabetes in rats. The present study aimed to elucidate the detailed molecular target of cinnamon in cultured adipocytes and epididymal adipose tissue of type 2 diabetes model mice. Two-dimensional gel electrophoresis was employed to determine the molecular target of cinnamon in adipocytes. The function of Acyl-CoA synthetase long-chain family-1 (ACSL1), a molecular target of cinnamon that was identified in this study, was further investigated in 3T3-L1 adipocytes using specific inhibitors. Type 2 diabetes model mice (KK-Ay/TaJcl) were used to investigate the effect of CE on glucose tolerance, ACSL1 expression, and related signal molecules in vivo. CE decreased ACSL1 mRNA and protein expression in 3T3-L1 adipocytes but increased glucose uptake and AMPK signaling activation; moreover, a similar effect was observed with an ACSL1 inhibitor. CE improved glucose tolerance and downregulated ACSL1 in mice adipose tissue in vivo. ACSL1 was demonstrated as a molecular target of CE in type 2 diabetes both in a cell culture system and diabetic mouse model.

## Introduction

There are approximately 371 million patients with diabetes mellitus worldwide, of which 4.8 million died due to diabetes-related diseases in 2017^1^. It has been predicted that in the next two decades, the numbers will double, and their direct medical costs will triple due to the combined effects of an increasingly aging population and higher rates of individuals that are overweight and obese^2^. Adipose tissue produces and secretes a variety of biologically active molecules called adipocytokines. The dysregulated production of adipocytokines, such as inflammatory cytokines in visceral fat in obesity, is involved in the development of abnormal glucose tolerance and insulin resistance^3–6^. Thus, therapeutic agents that target adipocytes to regulate energy metabolism and ameliorate insulin resistance have received considerable attention^7^.

Cinnamon has a long history as an important component in Chinese medicine. The extracts prepared from the bark of trees of the genus *Cinnamomum* have been prescribed for more than 2000 years in China, and their use in Chinese medicine was initially documented in Shen-Nong’s Herbal^8^. A number of in vitro and in vivo studies have demonstrated that cinnamon improves both insulin resistance and glucose metabolism^9–21^. However, the detailed mechanism of these antidiabetic properties has not yet been elucidated and is still controversial. Our previous study revealed that oral administration of a hot-water extract of cinnamon upregulates mitochondrial uncoupling protein-1 and enhances GLUT4 production and translocation in the muscle as well as GLUT4 translocation in the adipose tissues^15^. We further demonstrated the antidiabetic effects of cinnamon on the insulin and AMPK signaling pathways mediating glucose uptake in 3T3-L1 adipocytes and C2C12 myotubes^21^.

This study aimed to elucidate the molecular target of cinnamon in adipocytes. We identified Acyl-CoA synthetase long-chain family-1 (ACSL1) as a molecular target in the antidiabetic effect of cinnamon. ACSL1 has also been reported to regulate the incorporation of fatty acid into adipocytes^22–25^, inflammatory monocytes/macrophages, and the atherosclerosis in type 1 diabetes^26, 27^. Here we describe the function of ACSL1 in adipocytes as a molecular target of CE as well as in the amelioration of type 2 diabetes in a mice model in vivo*.*

## Results

### ACSL1 is identified as molecular target of cinnamon extract (CE) by two-dimensional gel electrophoresis (2DE) and LC–MS/MS analysis

**Figure 1:**
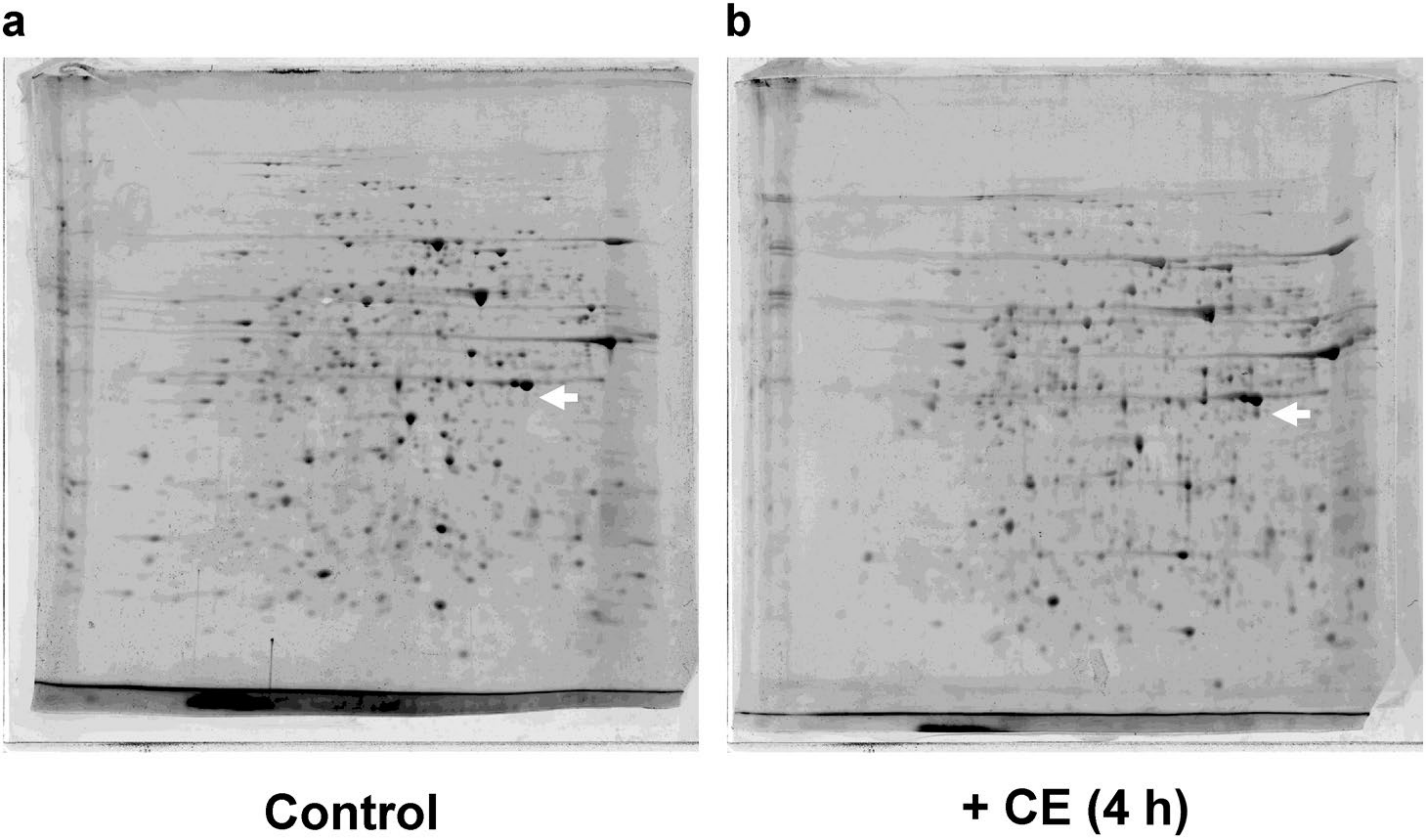
Two-dimensional gel electrophoresis of the total protein extracted from cinnamon extract treated 3T3-L1 adipocytes. Total proteins extracted from 3T3-L1 adipocytes were separated using two identical two-dimensional electrophoresis gels. (**a**) The full-length Flamingo Fluorescent Protein Gel staining patterns of untreated adipocytes and (**b**) adipocytes treated with 30 μg/mL of cinnamon extract (CE) for 4 h. n = 3/group.

The proteins from 3T3-L1 adipocytes treated with or without CE were subjected to 2DE analysis. A differentially expressed protein spot (Fig. 1a *vs.* b, arrow) could be reproducibly detected near 45 kDa. The changes in the differentially expressed spots, which were subjected to mass spectrometry identification, are presented in Supplementary Table S1. ACSL1 is constitutively different from the identical location of blank gel. The full-length images of the 2DE analyses for the three experiments are provided in Supplementary Fig. S1.

### CE decreased ACSL1 mRNA and protein expression in 3T3-L1 adipocytes

**Figure 2:**
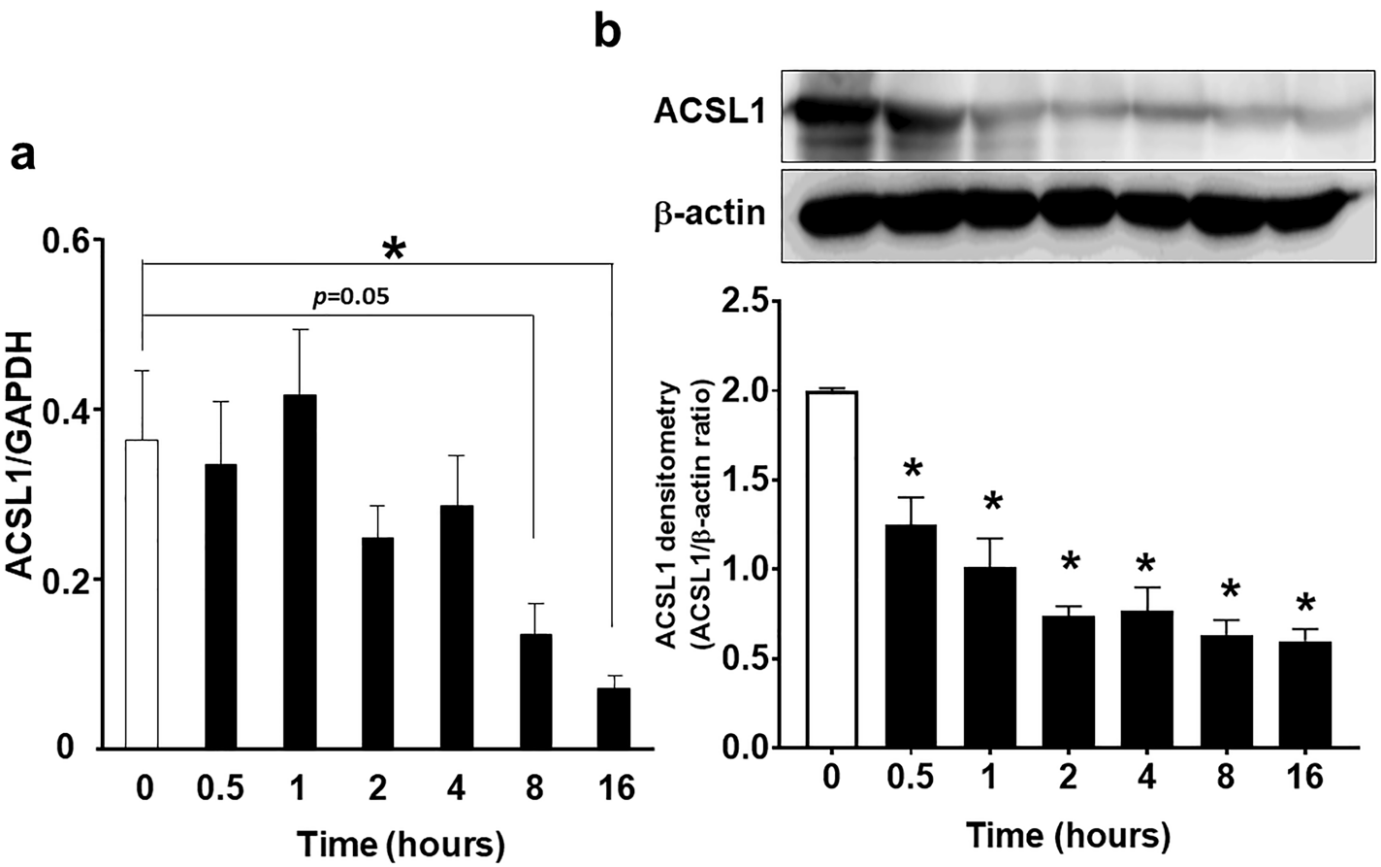
Cinnamon extract (CE) regulates Acyl-CoA synthetase long-chain family 1 (ACSL1) mRNA and protein expression in a time-dependent manner in 3T3-L1 adipocytes. 3T3-L1 adipocytes were serum-starved for 8 h in DMEM and then treated with 30 μg/mL of CE for 0.5–16 h. (**a**) Real-time PCR was employed to determine ACSL1 mRNA expression. (**b**) The levels of ACSL1 protein expression were measured via western blotting. The image is a cropped blot and the full-length images of the western blot for the three experiments are provided in Supplementary Fig. S2a. Each value represents the mean ± SD of three different experiments (n = 3/group). *p < 0.05, compared with the control values.

To elucidate the effect of CE on ACSL1, we employed differentiated 3T3-L1 adipocytes. CE (30 µg/mL) significantly decreased ACSL1 mRNA and protein expression in a time-dependent manner (Fig. 2a,b). The full-length images of the western blots for the three experiments are provided in Supplementary Fig. S2a.

### CE decreased lipogenesis and lipid accumulation mRNA expression and increased AMPK phosphorylation

**Figure 3:**
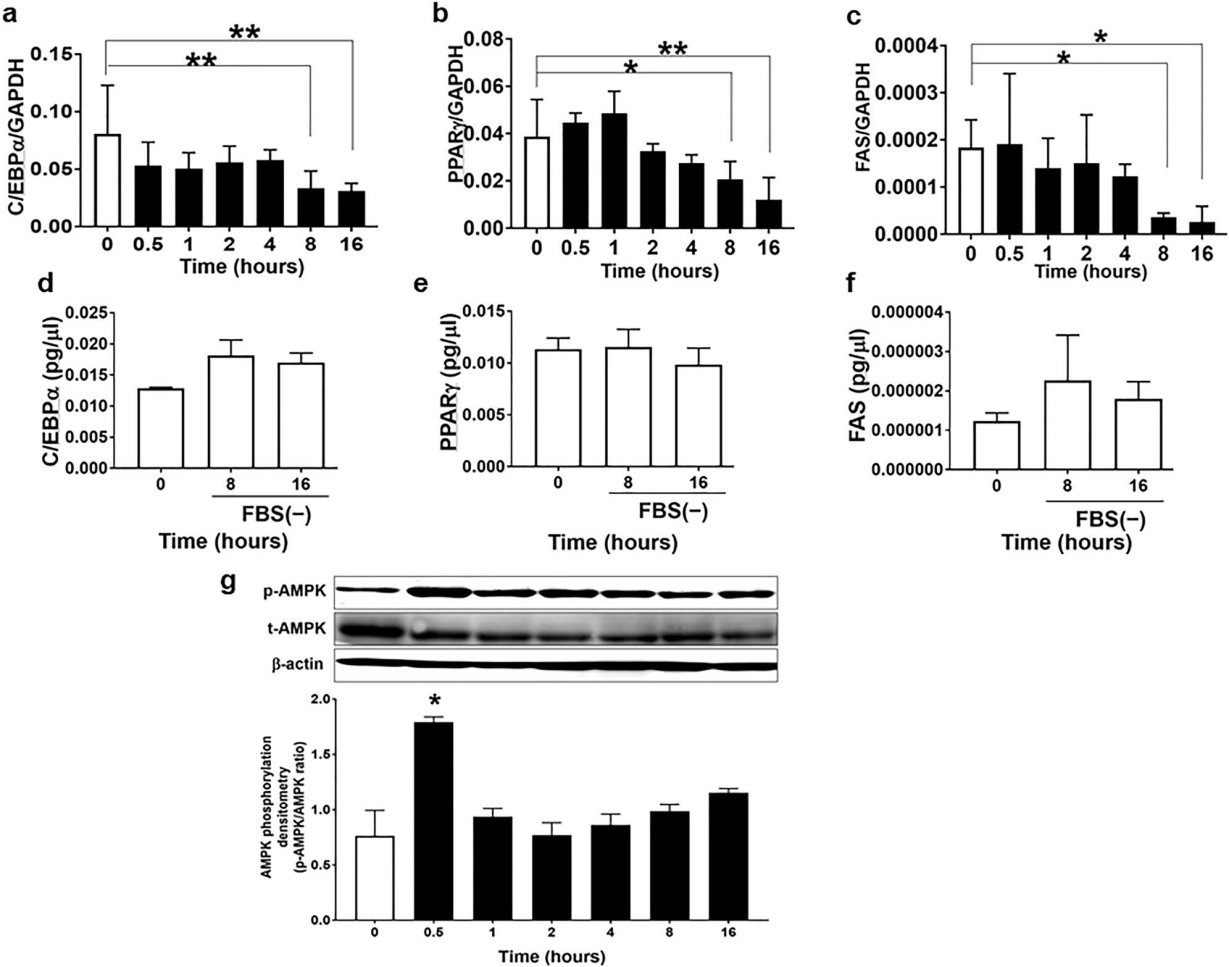
Cinnamon extract (CE) regulates lipogenesis marker expression in a time-dependent manner in 3T3-L1 adipocytes. (**a**–**d**) 3T3-L1 adipocytes were serum-starved for 8 h and then exposed to 30 μg/mL of CE for another 0.5–16 h. Real-time PCR was employed to determine mRNA expression of (**a**) C/EBPα, (**b**) PPARγ, and (**c**) FAS relative to GAPDH. Each value represents the mean ± SD of three different experiments (n = 3/group). *p < 0.05, **p < 0.01, compared with the control values. (**d**–**f**) 3T3-L1 adipocytes were serum-starved for 8 h and then maintained in serum-free culture for another 8–16 h. Real-time PCR was employed to determine the mRNA expression of (**d**) C/EBPα, (**e**) PPARγ, and (**f**) FAS. Each value represents the mean ± SD of three different experiments (n = 3/group). (**g**) 3T3-L1 adipocytes were serum-starved for 8 h and then exposed to 30 µg/mL of CE for another 0.5–16 h. The level of AMPK phosphorylation was measured via western blotting using total AMPK as loading controls. The image is a cropped blot and the full-length images of the western blot for the three experiments are provided in Supplementary Fig. S2b. In some images, different parts of the same membrane were cut out and reacted with different antibodies. The cutouts are clearly distinguished using yellow separator lines. Each value represents the mean ± SD of three different experiments (n = 3/group). *p < 0.05, compared with the control values.

ACSL1 plays a significant role in the regulation of lipid synthesis. As CE decreased both ACSL1 mRNA and protein expression, we next examined the effect of CE on the lipogenesis and lipid accumulation mRNA expression. CE significantly decreased C/EBPα, PPARγ, and FAS mRNA expression in a time-dependent manner (Fig. 3a–c). The time of serum-free conditions did not influence the expression of these mRNAs (Fig. 3d–f). In addition, CE stimulated AMPK phosphorylation in 3T3-L1 adipocytes (Fig. 3g). These results indicate that CE downregulated lipid synthesis by reducing ACSL1 and upregulated energy metabolism by phosphorylating AMPK. The images of full-length western blots of AMPK phosphorylation for the three experiments are provided in Supplementary Fig. S2b. We induced the differentiation of 3T3-L1 cells into adipocytes by simultaneously adding CE and differentiation-inducing factors to the culture medium for 5 days. The adipocyte area was not affected by the addition of CE to the culture medium (Supplementary Fig. S3). These results suggested that CE had no direct effect on adipocyte differentiation.

### Acetyl-CoA carboxylase (ACC) and Akt is activated by an ACSL1 inhibitor and CE

**Figure 4:**
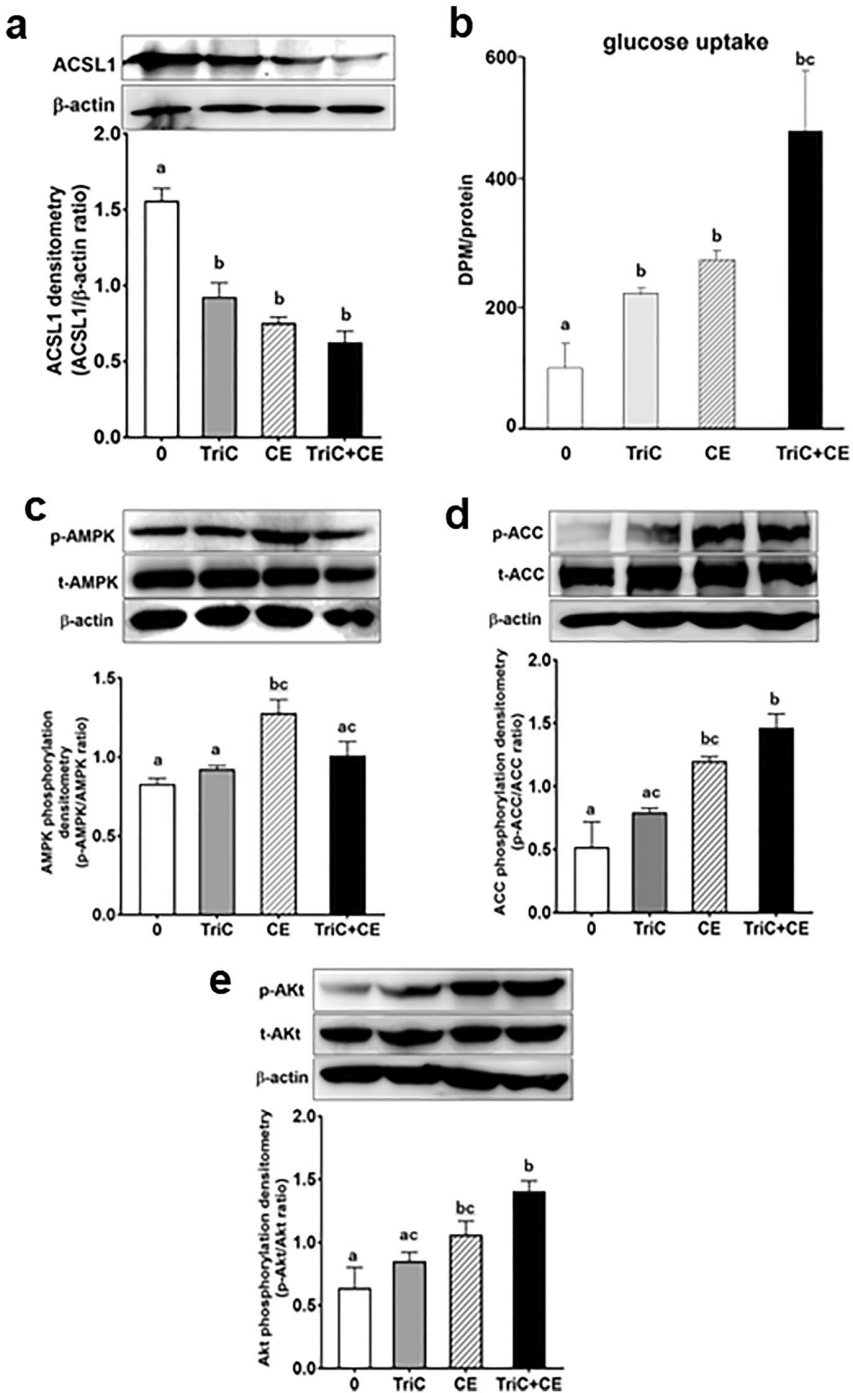
Triacsin C (TriC), an inhibitor of ACSL1, and Cinnamon extract (CE), regulate glucose uptake and ACC phosphorylation in 3T3-L1 adipocytes. 3T3-L1 adipocytes were serum-starved for 8 h in DMEM and then challenged with 10 µM TriC for 45 min or with 30 µg/mL of CE for 30 min. (**a**) ACSL1 expression levels were measured via western blotting. (**b**) The uptake of 2-deoxyglucose by the cells was assayed, as described in the Materials and Methods section. (**c**–**e**) The levels of AMPK, ACC, and Akt phosphorylation were measured via western blotting using total AMPK, ACC, and Akt as loading controls. Each value represents the mean ± SD of three different experiments (n = 3/group). Alphabet letters indicate intergroup comparisons. Different alphabet indicate statistically significant differences among groups (P<0.05). The image is a cropped blot and the full-length images of the western blot for the three experiments are provided in Supplementary Fig. S4 a–d. In Fig. S4d images, different parts of the same membrane were cut out and reacted with different antibodies. The cutouts are clearly distinguished using yellow separator lines.

To corroborate the role of ACSL1 in adipocytes, we examined the effect of an ACSL1 inhibitor. First, 3T3-L1 adipocytes were treated with the ACSL1 inhibitor Triacsin C (10 µM, TriC) to test its effects on 2-deoxyglucose uptake. TriC significantly decreased ACSL1 protein levels (Fig. 4a). CE and TriC significantly increased 2-deoxyglucose uptake into 3T3-L1 adipocytes (Fig. 4b), indicating the involvement of ACSL1 downregulation in glucose uptake. To investigate the molecular mechanism underlying the CE- and TriC-stimulated glucose uptake, we next studied the key kinases involved in the AMPK and insulin signaling pathways. The downregulation of ACSL1 induced AMPK phosphorylation that was accompanied by ACC (Ser^79^) phosphorylation (Fig. 4c,d); the ACC enzyme is located downstream of AMPK. TriC + CE significantly increased phosphorylation of ACC, but not that of AMPK (Fig. 4c,d). CE-induced Akt phosphorylation, and TriC did not exert action on the insulin pathway, and TriC + CE significantly increased phosphorylation of Akt vs. control or TriC group (Fig. 4e). These results indicate the involvement of the ACC and Akt pathway in the upregulation of CE-induced glucose uptake, through which ACSL1 is inhibited. The images of the full-length western blots of ACSL1 for the three experiments are provided in Supplementary Fig. S4a, whereas the images of the full-length western blot of AMPK, ACC, and Akt phosphorylation for the three experiments are provided in Supplementary Fig. S4b–d.

### CE treatment improved glucose tolerance and decreased ACSL1 protein expression in 2DM mice

**Figure 5:**
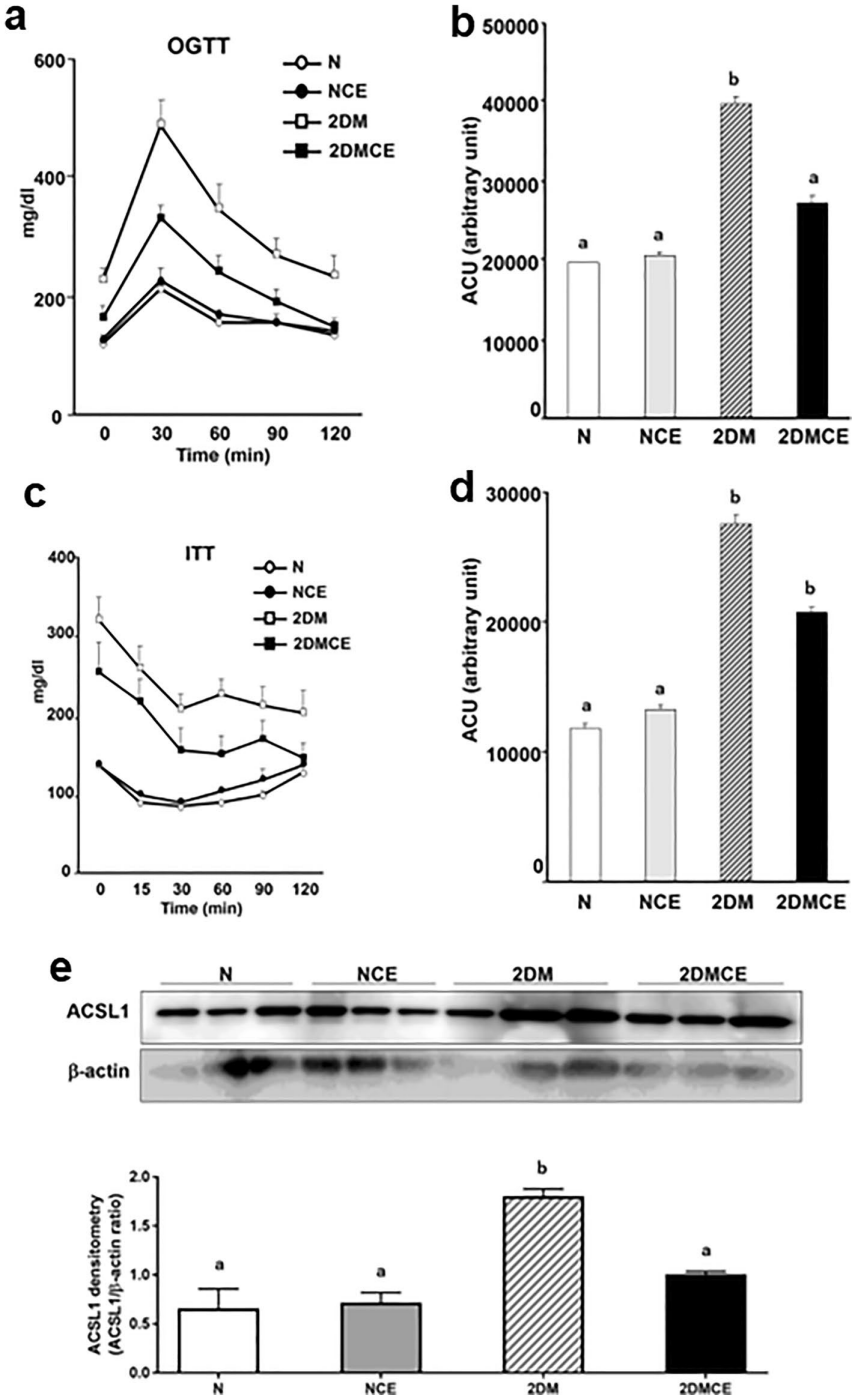
Cinnamon extract (CE) improves glucose intolerance and ACSL1 protein expression in type 2 diabetic mice. N: nondiabetic normal control group. Mice were orally administered 1-mL pure water for 8 weeks. NCE: CE-treated nondiabetic normal control group. CE (100 mg/kg bw/day) was orally administered for 8 weeks. 2DM: type 2 diabetes model mice group. Mice were orally administered 1-mL pure water for 8 weeks. 2DMCE: CE-treated type 2 diabetes model mice group. CE (100 mg/kg bw/day) was orally administered for 8 weeks. (**a**) For the oral glucose tolerance test, the mice fasted for 6 h prior to the test. Glucose (1.5 g/kg bw) was orally administered, and the blood glucose concentration was measured using the blood obtained via the tail vein at the indicated time points after the oral glucose loading. (**b**) The area under the curve (AUC) of the oral glucose tolerance test for panel (**a**). (**c**) For the insulin tolerance test, the rats fasted for 2 h. Then, insulin (0.75 U/kg bw) was intraperitoneally injected, and the blood glucose concentration was measured as previously described. (**d**) The AUC of the insulin tolerance test for panel c. Each value represents the mean ± SD of three different mice (n = 3/group). Alphabet letters indicate intergroup comparisons. Different alphabets indicate statistically significant differences among groups (P<0.01). (**e**) Western blot was employed to detect ACSL1 protein in the adipose tissue. The image is a cropped blot and the full-length images of the western blot are provided in Supplementary Fig. S5a and b. Each value represents the mean ± SD of three different mice (n = 3/group). Alphabet letters indicate intergroup comparisons. Different alphabets indicate statistically significant differences among groups (P<0.01).

To determine the effect of CE on type 2 diabetes, we conducted oral glucose tolerance test and insulin tolerance test in type 2 diabetes model mice in vivo. The administration of CE decreased blood triglycerides and insulin but did not demonstrate any effect on body weight (Table 1). In the oral glucose tolerance test, the CE-treated KK-Ay mice (2DMCE) exhibited lower blood glucose concentrations and area under the blood glucose concentration–time curve (ACU) compared with the untreated KK-Ay mice (2DM) at 30 min following glucose administration (Fig. 5a,b), indicating improved glucose tolerance, hyperlipidemia, and hyperinsulinemia in 2DM mice. Conversely, CE did not exhibit any effect on the blood glucose concentration of nondiabetic control mice (NCE) (Fig. 5a,b). The insulin tolerance test revealed that insulin sensitivity was not influenced by CE treatment in either the N or 2DM group (Fig. 5c,d). ACSL1 expression in the white adipose tissue of 2DM mice was increased compared with that of normal mice (N) and decreased compared with that of the CE-treated 2DM mice (Fig. 5e). The full-length images of the western blots are provided in Supplementary Fig. S5.

**Table 1:** Effects of cinnamon extract on body weight (bw) and plasma parameters.

Group	bw (g)	TC (mg/dL)	TG (mg/dL)	NEFA (mEq/L)	Adipo (ng/mL)	Insulin (ng/mL)
N	26.843 ± 1.18^a^	58.789 ± 7.96^a^	76.633 ± 17.91^a^	0.646 ± 0.00	1.972 ± 195	0.4167 ± 0.24^a^
NCE	24.100 ± 0.66^a^	82.456 ± 8.79^a^	91.022 ± 8.86^a^	0.751 ± 0.02	1.574 ± 1.56	0.1695 ± 0.03^a^
2DM	45.729 ± 1.99^b^	155.678 ± 36.95^b^	229.800 ± 31.62^b^	1.430 ± 0.16	9.637 ± 0.81	8.4123 ± 2.79^b^
2DMCE	44.300 ± 1.82^b^	120.677 ± 18.77^ab^	157.633 ± 21.30^c^	1.295 ± 0.09	8.237 ± 0.67	2.2831 ± 1.00^a^

Values are expressed as mean ± SD, n = 3/group.

The alphabetical symbols a, b, and c on the right shoulder of the number indicate intergroup comparisons. Different superscript letters indicate statistically significant differences among groups (*P* < 0.05).

N: nondiabetic normal control group. Mice were orally administered 1-mL pure water for 8 weeks. NCE: CE-treated nondiabetic normal control group. CE (100 mg/kg bw/day) was orally administered for 8 weeks.

*2DM* type 2 diabetes model mice group. Mice were orally administered 1-mL pure water for 8 weeks, *2DMCE* CE-treated type 2 diabetes model mice group. CE (100 mg/kg bw/day) was orally administered for 8 weeks, *bw* body weight, *TC* plasma total cholesterol content, *TG* plasma triglyceride content, *NEFA* plasma nonesterified fatty acid content, *Adipo* plasma adiponectin content, *Insulin* plasma insulin content.

## Discussion

We have demonstrated that the ACSL1 is a molecular target of CE for diabetes, both in cell subjects and mouse models of diabetes; CE specifically decreased ACSL1 levels in adipocytes. Such a decrease in ACSL1 expression was associated with stimulated glucose uptake and improved abnormal glucose tolerance in diabetic mice. In addition, we demonstrated that ACSL1 inhibition increases the activity of the AMPK signal associated with reduced lipogenesis, indicating that CE might represent properties as a hypoglycemic drug that does not initiate fat accumulation.

To identify the molecular target of the antidiabetic effect of CE, we used two-dimensional gel electrophoresis (2DE) analysis. It has been reported that the resolution of spots on strips with a narrow pH range (pH 4–7) is very high^28^, and O’Farrell et al. used the pH 5–7 and 3.5–10 in constant pH gradient^29^. We demonstrated good reproducibility of a strip of pH 5–8 and clearly visualized protein spots using Flamingo Fluorescent Protein Gel Stain (Fig. 1). A protein spot was chosen from the gels, and we identified a protein spot near 45 kDa following CE treatment. These proteins were either components of the cytoskeleton (β-actin, type 1 cytoskeletal 10), chaperones (complex protein), or proteases (Supplementary Table S1), which are the major actors required for cell survival in general. An exception is ACSL1, which is an essential factor for the synthesis of triacylglycerol. The expression of ACSL1 in CE-treated 3T3-L1 adipocytes might suggest the involvement of CE in energy metabolism. Next, we validated ACSL1 mRNA and protein expression in CE-treated 3T3-L1 adipocytes. Conversely, CE treatment reduced the expression of ACSL1 mRNA and protein. ACSL1 is typically found at 75–80 kDa on the Western blot; however, it is detected in the spot migrated at 45 kDa on 2DE, indicating that it contains fragmented proteins. As shown in Supplementary Table S1, other proteins such as actin were detected in excised spot on the 2DE gel. The fragmented protein and β-actin were separated together and detected at around 45 kDa.

ACSL1 has been reported to drive the incorporation of fatty acids in 3T3-L1 adipocytes and hepatic cells^25, 30–32^. Kanter et al. reported that the expression of ACSL1 mRNA in the monocyte of type 1 diabetes mouse models and patients was significantly increased compared to the healthy group. Furthermore, the ACSL1 deficiency model significantly reduced the release of proinflammatory cytokines and chemokines and prevented diabetic atherosclerosis^26^. In adipose tissue and liver, which are insulin target tissues, ACSL1 has been reported to be present in cell membranes, lipids, endoplasmic reticulum, and mitochondria^22, 33–36^. Regarding the expression of ACSL1 in each tissue, it has been reported that ACSL1 expression is highest in adipose tissue, especially during differentiation into adipocytes, where ACSL1 is markedly increased^37^. In ACSL1 knockdown experiments using 3T3-L1 adipocytes, the decrease in ACSL1 only increased lipolysis and did not affect fatty acid uptake^38^. These reports support our findings that CE suppressed lipid synthesis by decreasing the expression of ACSL1 (Fig. 3a–c). Our findings indicate that ACSL1 is an important factor in mediating the AMPK signal activation in adipocytes and in regulating glucose tolerance in diabetes. It has been reported that ACSL1 accounts for 80% of ACSL activity in adipose tissue^39, 40^. The possibility that other isoforms contribute to acyl-CoA synthesis to compensate for the decrease in ACSL1 protein cannot be ruled out. However, other isoforms of ACSL have been reported to play important roles in lipid metabolism in the liver and muscle^41, 42^. In our next study, we will investigate the effects of cinnamon on other isoforms of ACSL in the liver and muscle.

Jessica et al. found that mice with an adipose tissue-specific knockout of ACSL1 (Acsl1A-/-) did not develop lipodystrophy, even though the rate of fatty acid (FA) oxidation was significantly reduced compared to adipocytes in control mice. Similarly, body weight increased to the normal range, and there was no difference in adipocyte size between the Acsl1A−/− and control group^39^. These data are consistent with the results of our experiments, in which cinnamon administration had no effect on the body weight of mice, although it significantly reduced the expression of ACSL1 in adipose tissue. However, these studies did not examine the relationship between ACSL1 expression and glucose metabolism. As this study focused on the ameliorative effects of cinnamon extract on glucose intolerance, we also plan to elucidate its effects on lipid metabolism in future studies.

**Figure 6:**
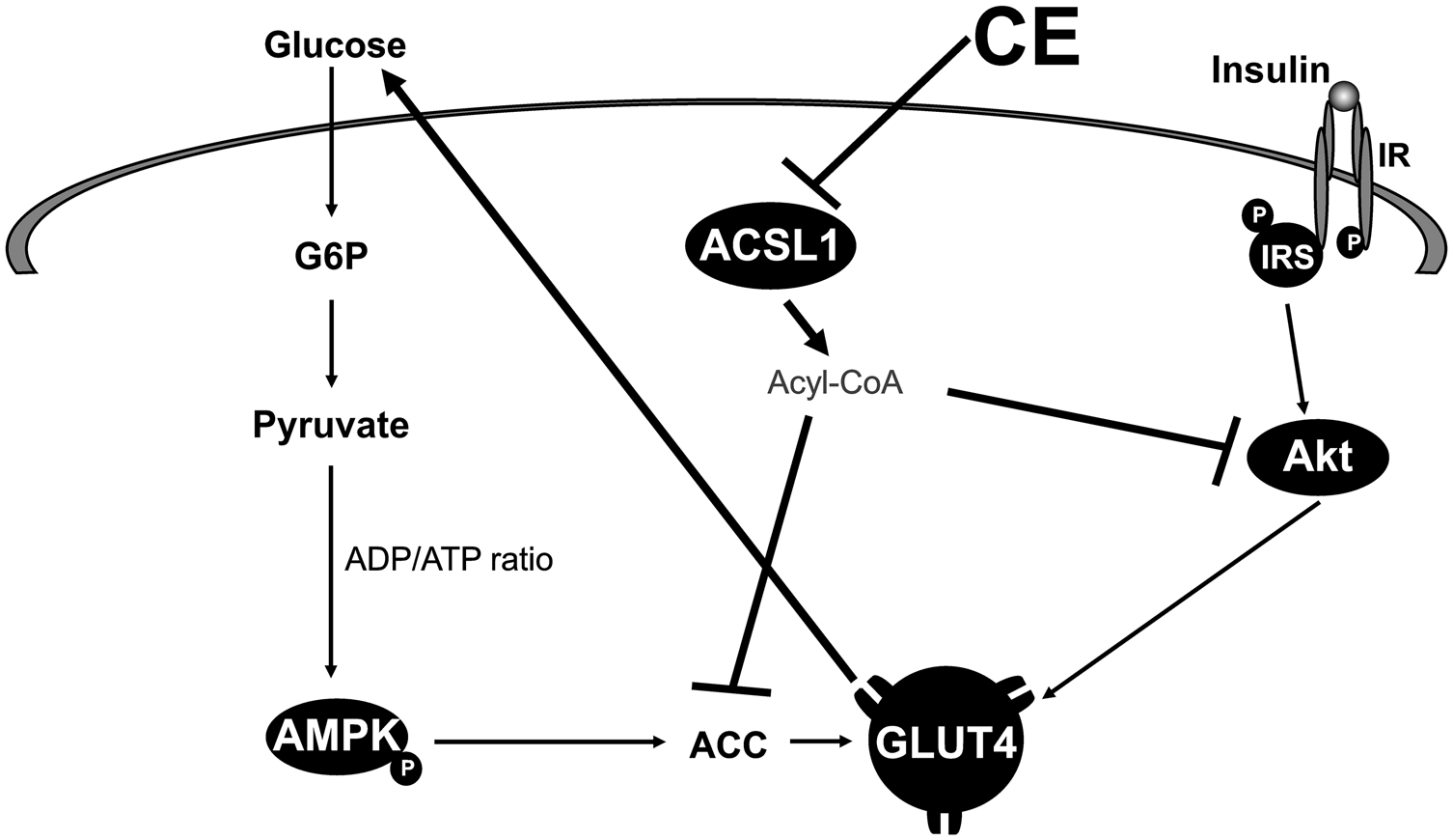
Outline of the mechanisms for the stimulation of glucose metabolism by cinnamon. Using 3T3-L1 adipocytes, we demonstrated that CE downregulates ACSL1 to activate the AMPK signaling pathway that stimulates glucose uptake and suppresses lipogenesis.

In this study, we have demonstrated for the first time that glucose uptake and the improvement of abnormal glucose tolerance are initiated by reduced ACSL1 expression in adipocytes (Figs. 4b and 5a). To elucidate the relationship between upregulated glucose transport and ACSL1 suppression, we used the known ACSL1 inhibitor Triacsin C^43–45^. In fact, we confirmed that the inhibition of ACSL1 and stimulation of glucose uptake were mediated by increased AMPK-ACC phosphorylation (Fig. 4c,d). Almouhanna and Hardie et al. reported that when the intracellular carbohydrate supply cannot keep pace with the demand for ATP, it increases the ADP/ATP ratio, followed by a larger increase in the AMP/ATP ratio, which activates the AMPK signaling pathway^46, 47^. CE may promote AMPK phosphorylation by increasing the ADP/ATP ratio in cells, although further studies are required to confirm this possibility. Coupled with a fall in cytoplasmic acetyl-CoA due to a restricted carbohydrate supply, fatty acids become the main fuel for ATP production^47^. Coincidentally, ACSL1 inhibition decreases fatty acid uptake into cells due to suppressed triacylglycerol synthesis, whereupon due to the depletion of energy sources, GLUT4 will translocate to the plasma membrane (Fig. 6). CE increased glucose uptake, concurrently reduced lipogenesis/lipid accumulation mRNA expression in culture 3T3-L1 adipocyte, and reduced plasma triglyceride content in type 2 diabetic mice (Figs. 3 and 4b, Table 1). These data were supported by the superimposed speculation that CE increases glucose uptake in adipocytes and simultaneously inhibits lipid accumulation. Although there appears to be a synergistic effect between CE and TriC on glucose uptake (Fig. 4b), there was no significant difference between the TriC + CE group and the group with CE or TriC alone. Regarding the mechanism of cinnamon action, ACSL1 may not explain the whole mechanism, but in this study, the expression of ACSL1 protein was decreased by cinnamon addition, and inhibition of this factor promoted glucose uptake, suggesting that ACSL1 is a key factor in cinnamon action. However, owing to the limited scope of this study, we were unable to examine all glucose uptake-related signals, and the effects of cinnamon on other signals should be further investigated in future studies. In this study, both CE and TriC reduced ACSL1 and consistently increased glucose uptake. However, only CE increased AMPK and ACC phosphorylation, whereas TriC did not alter these proteins. Although the western blot analysis showed a trend toward increased phosphorylation of these proteins, the quantified values of the protein bands showed no significant differences. Thus, TriC had no significant effect on AMPK and ACC phosphorylation, which was likely a limitation of our experimental system. A higher concentration of TriC may be required in the culture system to obtain obvious changes in the phosphorylation that would be detectable through western blot analyses. Nevertheless, the concentration of TriC used in this study may be useful for future studies as a reference concentration that promotes glucose uptake in cultured adipocytes.

Finally, we observed that CE improved glucose intolerance but not insulin resistance in type 2 diabetic mice (Fig. 5). This observation is consistent with the antidiabetic effect of CE on insulin-uncontrolled type 1 diabetic rats that we previously reported^15^. Compared with that of normal mice, the expression of ACSL1 in white adipose tissue was increased in 2DM mice and decreased in CE-treated 2DM mice (Fig. 5c). This data strongly suggests that ACSL1 is critically important in mediating abnormal glucose tolerance in the adipose tissue of type 2 diabetes. Furthermore, CE treatment of 2DM mice significantly decreased plasma insulin concentrations compared with untreated 2DM mice (Table 1). Glucose uptake of 3T3-L1 adipocytes incubated with no reagent (control group), 100 nM insulin, or 30 µg/mL CE for 30 min was examined. Glucose uptake significantly increased in the CE and insulin groups (Supplementary Fig. S6). These data suggest that cinnamon promotes glucose uptake as well as insulin. Based on the above, the data strongly supports the idea that CE, rather than insulin, promotes glucose uptake in peripheral tissue.

To identify the active compound in CE, we performed analyses with high-performance liquid chromatography in our previous studies and identified two compounds from CE; 1 mg CE contained 8.5 μg of cinnamaldehyde and 3.6 μg of cinnamyl alcohol^15^. Two compounds and a mixture of unknown components were shown to play a significant role in stimulating glucose uptake in adipocytes (data not shown). However, long-term research is still important to determine candidate active compounds and their detailed pharmacological properties. Thus, we will investigate these aspects in future studies. We believe that it is important to understand the mechanism through which CE decreases ACSL1 expression. A direct interaction between one of the components of the extract and the enzyme may inhibit biosynthesis but may also be involved in the regulation of mRNA degradation. Future studies should examine the mechanism by which CE decreases ACSL1.

In conclusion, the current study demonstrated for the first time that CE decreases ACSL1 expression to increase glucose uptake in 3T3-L1 adipocytes and 2DM mice. As insulin resistance has been associated with the progressive development of type 2 diabetes mellitus, natural compounds that reduce ACSL1 due to a stimulated AMPK signal should have a significant clinical impact on type 2 diabetes mellitus.

## Methods

### Preparation of CE

CE was prepared by the method described previously^15^. Cinnamon (*Cinnamomum zeylanicum*) was a gift from House Foods Corporation (Tokyo). The sticks (250 g) were soaked in 2500 mL of water for 24 h at room temperature (RT) and then heated for 30 min at 100 °C. The CE was lyophilized, and the powder was stored at − 20 °C until further use^15^. This study complies with relevant institutional, national, and international guidelines and legislation.

### Reagents

The primary antibodies against phosphorylated (p)-Akt (Ser473), p-ACC (Ser79), p-AMPK (Thr172), Akt, ACC, AMPK, and ACSL1 were purchased from Cell Signaling Technology (Tokyo, Japan). Anti-β-actin was obtained from Sigma-Aldrich (MO, USA). Horseradish peroxidase-conjugated secondary antibodies were purchased from Jackson ImmunoResearch Laboratories (PA, USA), and Triacsin C was obtained from Enzo Life Sciences (NY, USA).

### Cell culture

The 3T3-L1 fibroblasts were obtained from Health Science Research Resources Bank (Osaka, Japan), which is the same sources as previously described^48^. 3T3-L1 fibroblasts were cultured and induced to differentiate into adipocytes using the method described previously^48^.

### Two-dimensional gel electrophoresis (2DE) and SDS-PAGE

Cultured 3T3-L1 adipocytes were collected and homogenized in lysis buffer [5 M urea, 2 M thiourea, 20% CHAPS, 65-mM DTT, and 2% Pharmalyte (pH 5–8)] with sonication for 5 min at 3 kHz/130 W (UCD-130TM, Cosmo Bio, Tokyo) on ice and then centrifuged at 760×*g* for 5 min. Isoelectric focusing was performed using a Cool Phore Star IPG-IEF (Anatech, Japan) with Immobiline DryStrips (18 cm, pH 5–8; Bio-Rad, USA). The same wet weight sample application piece (Anatech, Japan) was placed on the gel 1 cm from the cathodic end. The isoelectric focusing voltage was as follows: 500 V (2 h), 700 V (1 h), 1000 V (1 h), 1500 V (1 h), 2000 V (1 h), 2500 V (1 h), 3000 V (1 h), and then 3500 V (10 h). The electric power supply was maintained at 500 V after 10 h at 3500 V. After electrophoresis, the gel strip was treated with sodium dodecyl sulfate (SDS) (6 M urea, 42-mM DTT, 0.5 M Tris–HCl (pH 6.8), 10% (w/v) SDS, 0.1% (w/v) BPB, and 50% (v/v) glycerol) for 30 min at RT with agitation. Then, the gel strip was incubated for 15 min at RT in alkylation buffer (6 M urea, 0.3 M iodoacetamide, 0.5 M Tris–HCl (pH 6.8), 10% (w/v) SDS, 0.1% (w/v) BPB, and 50% (v/v) glycerol). The alkylated gel strip was subjected to SDS-PAGE, and electrophoresis was performed under a constant current (5 mA per gel) until the dye front of BPB approached the gel bottom. The protein on the 2D gel slab was stained with Flamingo Fluorescent Protein Gel Stain (Bio-Rad, USA). The 2D gel image was obtained from Storage Phosphor Screen with Typhoon 9410 (GE Healthcare, Japan).

### Measurement of glucose uptake

Glucose uptake was measured using the method described by Ragolia et al.^49^. Briefly, 3T3-L1 adipocytes cultured in 60-mm dishes (Becton Dickinson, NT, USA) were serum-starved in Dulbecco’s Modified Eagle Medium (DMEM) for 16 h. Subsequently, the cells were washed three times with phosphate buffered saline (pH 7.4) and then incubated for 30 min in the following medium: DMEM alone, DMEM containing either 100 nM insulin or 30 µg/mL of CE, or a combination of both insulin and CE. Then, 0.5-mM 2-deoxy-d-[2,6-^3^H]-glucose (1.5 µCi/well, Moravek Biochemicals, CA, USA) was added to the cells and incubated for 15 min. Finally, the cells in triplicate were washed four times with phosphate buffered saline containing 0.3-mM phloretin and lysed in 1 mL of 1 M NaOH for scintillation counting.

### Western blot analysis

Western blot analysis was performed using the method described previously^21^. Whole-cell lysates and plasma membrane fractions were subjected to SDS-PAGE, and the proteins that had migrated were electrically transferred to a microporous polyvinylidene fluoride membrane (Millipore Corporation, MA, USA) for western blotting^21^. The membrane was incubated at 4 °C for 18 h with antibodies against the following proteins: p-Akt, Akt, p-AMPK, AMPK, p-ACC, ACC (all at 1:1000), and β-actin (1:2000)^21^. After incubation, anti-mouse (1:2000 for β-actin) or anti-rabbit IgG horseradish peroxidase conjugate (1:2000 for p-Akt, Akt, p-AMPK, AMPK, p-ACC, and ACC) was added, and the membrane was incubated for 30 min at RT. The antigenic proteins on the membrane were visualized via chemiluminescence using a Lumi-Light^PLUS^ Western Blotting Kit (Roche Diagnostics Co., Basel, Switzerland), and the images were evaluated using an Image Analyzer LAS-4000 (Fujifilm Co. Tokyo, Japan)^21^.

### RNA isolation and real-time PCR

Total RNA was extracted from 3T3-L1 adipocytes with the Isogen reagent (Wako Pure Chemical Inc., Osaka, Japan). Real-time reverse transcription–PCR was performed via the fluorescent dye SYBR Green I method using SYBR Premix Ex Taq and Perfect Real Time (Takara Bio, Shiga, Japan) with a StepOne Real-Time PCR system (Applied Biosystems, CA, USA). The primers used in this study were designed based on the GenBank™ information and synthesized by Invitrogen (Carlsbad, CA, USA). The PCR primers used for ACSL1, PPARγ, C/EBPα, FAS, and GAPDH are presented in Supplementary Table S2.

### Animal experiments

Four-week-old male KK-Ay/TaJcl mice, a model of type 2 diabetes mellitus (2DM), and C57BL/6J, a nondiabetic normal control (N), were purchased from CLEA Japan Inc. (Tokyo, Japan), and housed individually in a stainless steel, wire-bottomed cage in a temperature-controlled room (22 °C–23 °C) with a 12-h photoperiod. The mice were provided a pellet diet (CE-2, CLEA Japan Inc., Tokyo, Japan) and water ad libitum. After 1 week of acclimation, the mice were divided into two groups with matched body weight: (1) the control group without cinnamon (N, 2DM) and (2) the cinnamon-treated group (NCE, 2DM + CE). Regarding the nondiabetic normal control group (N), mice were orally administered 1 mL pure water for 8 weeks and the CE-treated nondiabetic normal control group (NCE), CE (100 mg/kg bw/day) was orally administered for 8 weeks. For type 2 diabetes model mice group (2DM), mice were orally administered 1 mL pure water for 8 weeks. Regarding the CE-treated type 2 diabetes model mice group (2DM + CE), CE (100 mg/kg bw/day) was orally administered for 8 weeks. All experiments were conducted in accordance with the National Institutes of Health Guide for the Care and Use of Laboratory Animals and were approved by the Nihon University Animal Care and Use Committee (approval number AP11B086). Also, we confirm that all methods are reported in accordance with ARRIVE guidelines (https://arriveguidelines.org).

### Oral glucose tolerance test and insulin tolerance test

Oral glucose tolerance test and insulin tolerance test were performed using the method described previously^21^. Briefly, mice were orally administered CE (100 mg/kg bw/day) for 8 weeks. For the glucose tolerance test, the mice fasted for 6 h and were then orally administered glucose (1.2 g/kg bw)^21^. Blood glucose concentration was measured by DEXTER-Z II (Bayer Medical Co., Ltd., Leverkusen, Germany) using blood collected via the tail vein. For the insulin tolerance test, the mice fasted for 2 h and were then intraperitoneally administered insulin (0.75 U/kg bw)^21^. Then, blood glucose concentration was measured as described above^21^. Plasma insulin concentrations were assayed using ELISA Kit (Shibayagi, Gunma, Japan)^21^.

### Measurement of blood parameters and adipose tissue

Plasma total cholesterol, triglycerides, nonesterified fatty acids, and adiponectin concentrations were determined using commercial assay kits (Wako Pure Chemical). Epididymal adipocytes were homogenized using a Dounce homogenizer in ice-cold sucrose-Tris-ethylene glycol buffer (pH 7.4) containing 250-mM sucrose, 5-mM Tris–HCl, 2-mM ethylene glycol tetraacetic acid, and protease inhibitor mixture (Sigma-Aldrich). The homogenate was centrifuged at 800×*g* for 3 min at 4 °C to remove the tissue debris, and the supernatant that contained adipose tissue cytosol was collected to assay the protein contents.

### Statistical analysis

Statistical analysis was conducted using GraphPad Prism 7.0 (GraphPad Software, San Diego, CA). The results were expressed as mean ± SD. Each value represents the average of three different experiments (n = 3/group). We conducted statistical analysis via one-way ANOVA, followed by Dunnett’s test for multiple comparisons among several groups in Fig. 2, Fig. 3 and Supplementary Fig. S6. Pairwise comparisons were performed using Student’s t-test in the Supplementary Fig. S3. We conducted statistical analysis via two-way ANOVA, followed by Tukey’s test for multiple comparisons among several groups in Fig. 4, Fig. 5 and Table 1. Differences were considered significant at *p* < 0.05.

## Supplementary Information

Supplementary Information 1.

Supplementary Figure S1.

Supplementary Figure S2.

Supplementary Figure S3.

Supplementary Figure S4.

Supplementary Figure S4.

Supplementary Figure S5.

Supplementary Figure S6.

## Data Availability

All data generated and analyzed during this study are included in this published article.
