# Supplementary material for: Cinnamon extract improves abnormalities in glucose tolerance by decreasing Acyl-CoA synthetase long-chain family 1 expression in adipocytes
Source: Sci Rep. 2022 Jul 22;12:12574. doi: 10.1038/s41598-022-13421-9 (PMC9307619; doi:10.1038/s41598-022-13421-9)
Supplement: Supplementary file 1 — Supplementary Information 1. [file 41598_2022_13421_MOESM1_ESM.docx]

**Cinnamon extract improves abnormalities in glucose tolerance by decreasing Acyl-CoA synthetase long-chain family 1 expression in adipocytes**

Tsubame (Yan) Nishikai-Shen^1,2,3^_,_ Tomomi Hosono-Fukao^1^, Toyohiko Ariga^1^, Takashi Hosono^1^, Taiichiro Seki^1^*

^1^Department of Chemistry and Life Science, Nihon University College of Bioresource Sciences, Nihon University Graduate School of Bioresource Sciences, Kanagawa 252-0880, Japan

^2^Intractable Disease Research Center, Juntendo University School of Medicine, Tokyo 113-8421, Japan

^3^Division of Regenerative Therapy, Juntendo University Graduate School of Medicine, Tokyo 113-8421, Japan

* Corresponding author: Taiichiro Seki

Department of Chemistry and Life Science, Nihon University College of Bioresource Sciences, Nihon University Graduate School of Bioresource Sciences, Kanagawa 252-0880, Japan

Telephone No.: +81-466-84-3949

Fax No.: +81-466-84-3949

Email Address: [seki.taiichirou@nihon-u.ac.jp](mailto:seki.taiichirou@nihon-u.ac.jp)

**Supplementary Fig. S1** The full-length image of gel of all two-dimensional electrophoresis from 3T3-L1 adipocytes treated by CE. (a, c, e) The Flamingo Fluorescent Protein Gel staining patterns of untreated adipocytes and (b, d, f) adipocytes treated with 30 μg/mL of cinnamon extract (CE) for 4 h. n=3/group.

**Supplementary Fig. S2** The full-length images of western blot of protein expressed by 3T3-L1 adipocytes treated with CE. 3T3-L1 adipocytes were serum-starved for 8 h in DMEM and then incubated with 30 μg/mL of CE for 0.5–16 h. (a) ACSL1 protein expression. (b) Phosphorylated AMPK and AMPK protein expressions. In some images, different parts of the same membrane were cut out and reacted with different antibodies. The cutouts are clearly distinguished using yellow separator lines.

**Supplementary Fig. S3** The effect of CE on adipocyte differentiation. CE and differentiation-inducing factor were added to the culture for 5 days. (a) Oil red staining of the 3T3-L1 adipocyte on day 5 after the induction of differentiation. The upper photos show the group to which the differentiation- inducing factor was added (0 group), and the lower photos show the cells cultured with both the differentiation-inducing factor and CE (CD group). (b) The area of the adipocytes was measured. The area of adipocytes was not affected by the addition of CE to the culture. Each value represents the mean ± SD of three different experiments (n=3/group).

**Supplementary Fig. S4** The full-length images of western blot of protein expressed by 3T3-L1 adipocytes treated with CE and TriC. 3T3-L1 adipocytes were serum-starved for 8 h in DMEM and then incubated with 10 μM TriC for 45 min or with 30 μg/mL of CE for 30 min. (a) ACSL1 protein expression. (b-d) Phosphorylated AMPK, ACC, and Akt proteins measured with total AMPK, ACC, and Akt as loading controls. In some images, different parts of the same membrane were cut out and reacted with different antibodies. The cutouts are clearly distinguished using yellow separator lines.

**Supplementary Fig. S5** Western blot was employed to detect ACSL1 protein in the adipose tissue. N: nondiabetic normal control group. Mice were orally administered 1-mL pure water for 8 weeks. NCE: CE-treated nondiabetic normal control group. CE (100 mg/kg bw/day) was orally administered for 8 weeks. 2DM: type 2 diabetes model mice group. Mice were orally administered 1-mL pure water for 8 weeks. 2DMCE: CE-treated type 2 diabetes model mice group. CE (100 mg/kg bw/day) was orally administered for 8 weeks. The full-length images of Western blot were shown. (a) ACSL1 protein expression. (b) β-actin protein expression.

Supplementary Fig. S6 The effect of insulin and CE on glucose uptake. The uptake of 2-deoxyglucose by the cells was assayed, as described in the Materials and Methods section. The glucose uptake of 3T3-L1 adipocytes incubated with no reagent (control group), 100 nM insulin or 30 ug/ml CE for 30 min was examined. The glucose uptake was significantly increased in the CE group as well as the control group. These data suggest that cinnamon promotes glucose uptake as well as insulin. Each value represents the mean ± SD of three different experiments (n=3/group). ** p < 0.01, compared with the control values.

**Supplementary Table S1.** Differentially expressed protein identified via LC-MS/MS analysis along with specific manifestation of spots in two-dimensional gel electrophoresis

| Accession | Description | Score (%) | Coverage (%) | MW (kDa) |
| --- | --- | --- | --- | --- |
| 6671509 | actin, beta, cytoplasmic (*Mus musculus*) | 34.90 | 35.47 | 41.7 |
| 94391959 | PREDICTED: similar to Keratin, type I cytoskeletal 10 (*Mus musculus*) | 11.04 | 6.63 | 106.6 |
| 31560705 | acyl-CoA synthetase long-chain family member 1 (*Mus musculus*) * | 9.77 | 5.87 | 77.9 |
| 47059013 | type II keratin Kb36 (*Mus musculus*) | 3.94 | 3.90 | 58.9 |
| 94420504 | PREDICTED: similar to protease, serine, 3 (*Mus musculus*) | 2.98 | 10.20 | 10.7 |
| 6678643 | keratin complex 2, basic, gene 1 (*Mus musculus*) | 2.72 | 1.75 | 65.2 |
| 46275808 | keratin complex 2, basic, gene 17 (*Mus musculus*) | 2.70 | 1.27 | 70.9 |

**Supplementary Table S2.** PCR primers for the amplification of cDNAs

| Gene | Sequence |
| --- | --- |
| ACSL1 | 5'-TACTACTACGACGATGTCAGAACCA-3'-CCTCTTTGTAGGAAATCCACTCATA-5' |
|  |  |
| PPARγ | 5'-GCCCTTTGGTGACTTTATGG-3'-GCAGCAGGTTGTCTTGGATG-5' |
|  |  |
| FAS | 5'-TGATTAGCCTAAGACTGAAGCATCT-3'-CAAGTTTATAAATCTGCTCCCTTGA-5' |
|  |  |
| C/EBPα | 5'-GCAAAGCCAAGAAGTCGGTG-3'-AGGCGGTCATTGTCACTGGT-5' |
|  |  |
| GAPDH | 5'-TGTGTCCGTCGTGGATCTG-3'-GAGACAACCTGGTCCTCAGTG-5' |
|  |  |
