# Supplementary figures and images for: Cinnamon extract improves abnormalities in glucose tolerance by decreasing Acyl-CoA synthetase long-chain family 1 expression in adipocytes
Source: Sci Rep. 2022 Jul 22;12:12574. doi: 10.1038/s41598-022-13421-9 (PMC9307619; doi:10.1038/s41598-022-13421-9)

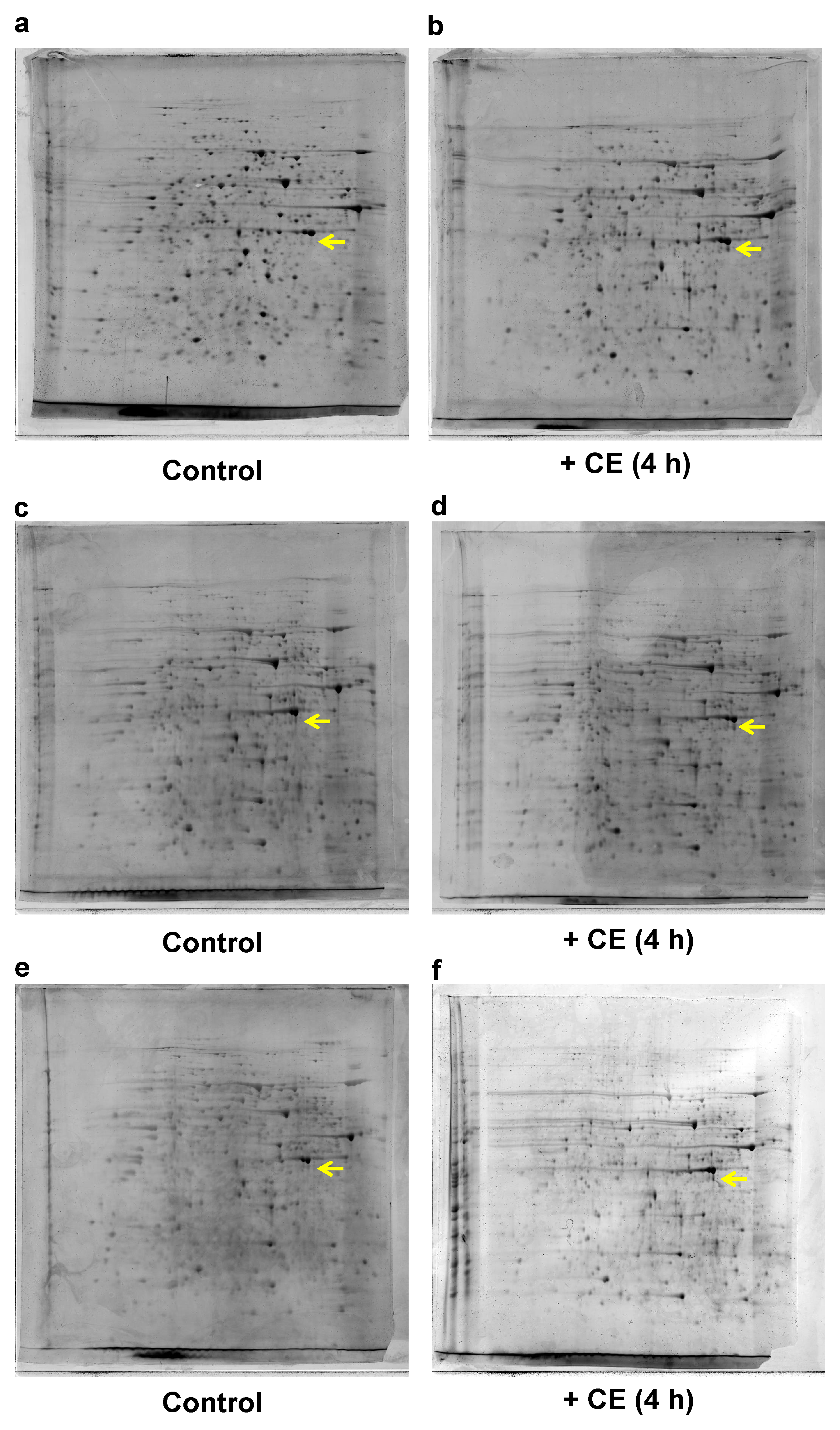

Supplement: Supplementary file 2 — Supplementary Figure S1. [file 41598_2022_13421_MOESM2_ESM.tif]

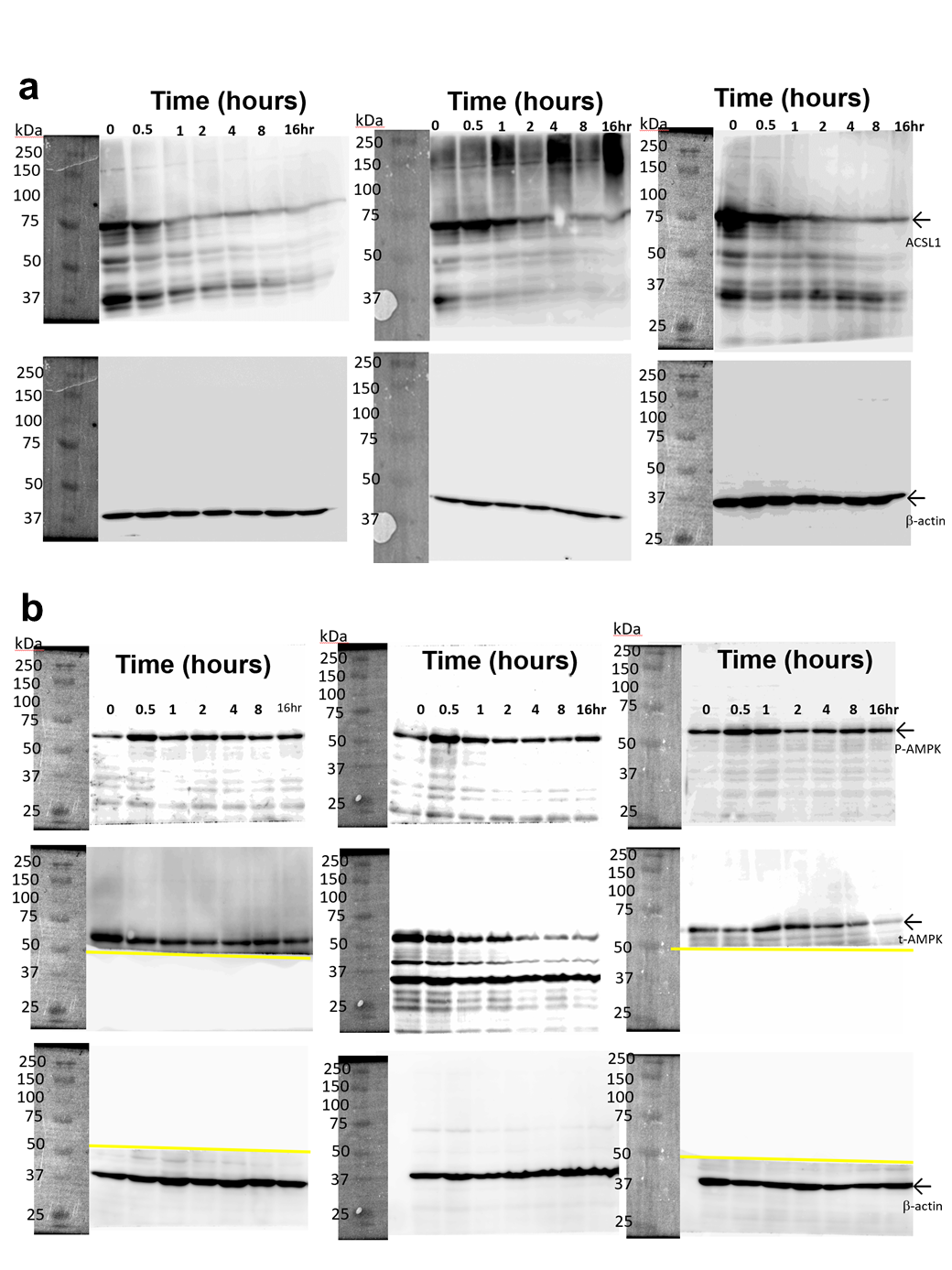

Supplement: Supplementary file 3 — Supplementary Figure S2. [file 41598_2022_13421_MOESM3_ESM.tif]

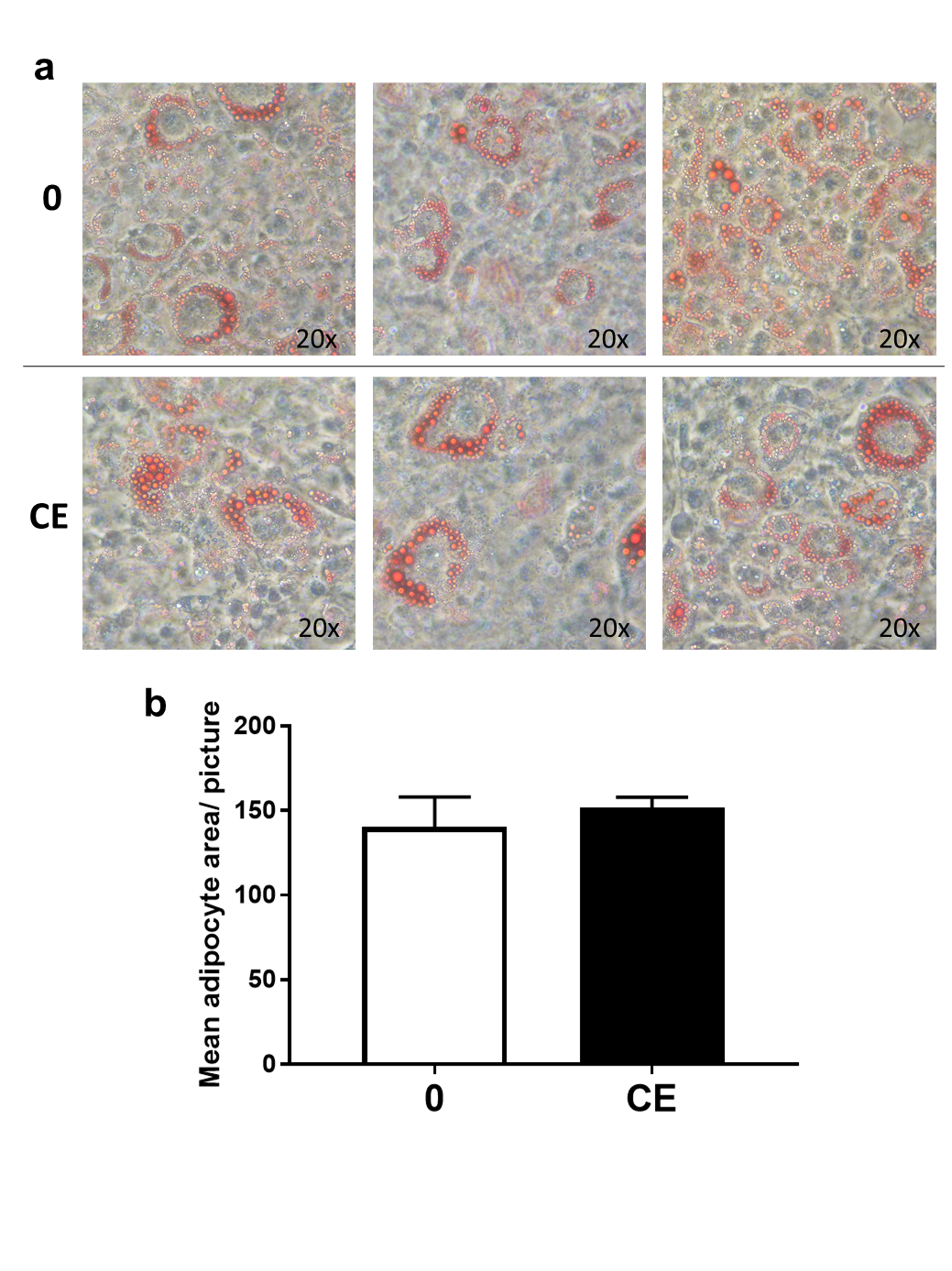

Supplement: Supplementary file 4 — Supplementary Figure S3. [file 41598_2022_13421_MOESM4_ESM.tif]

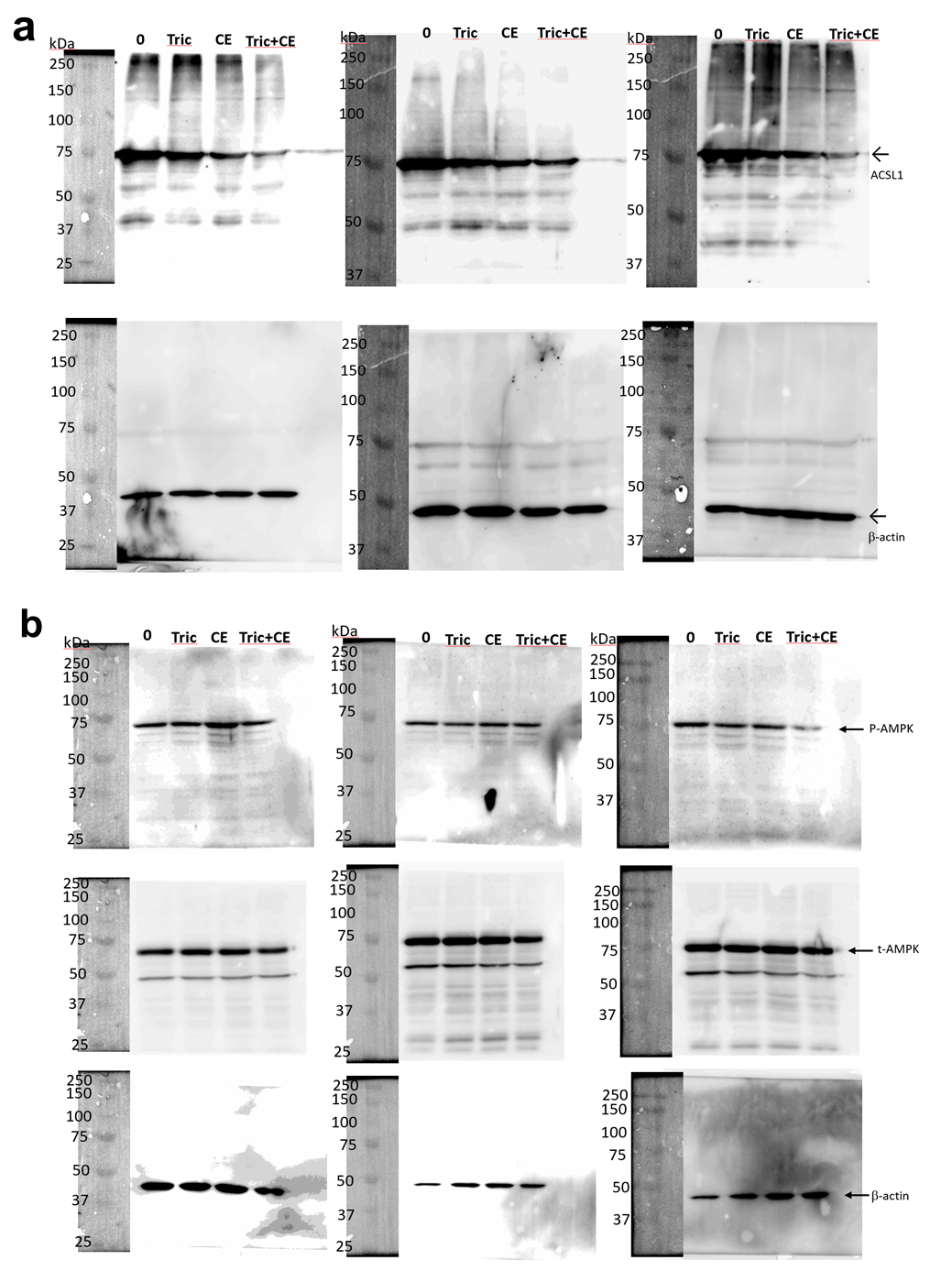

Supplement: Supplementary file 5 — Supplementary Figure S4. [file 41598_2022_13421_MOESM5_ESM.tif]

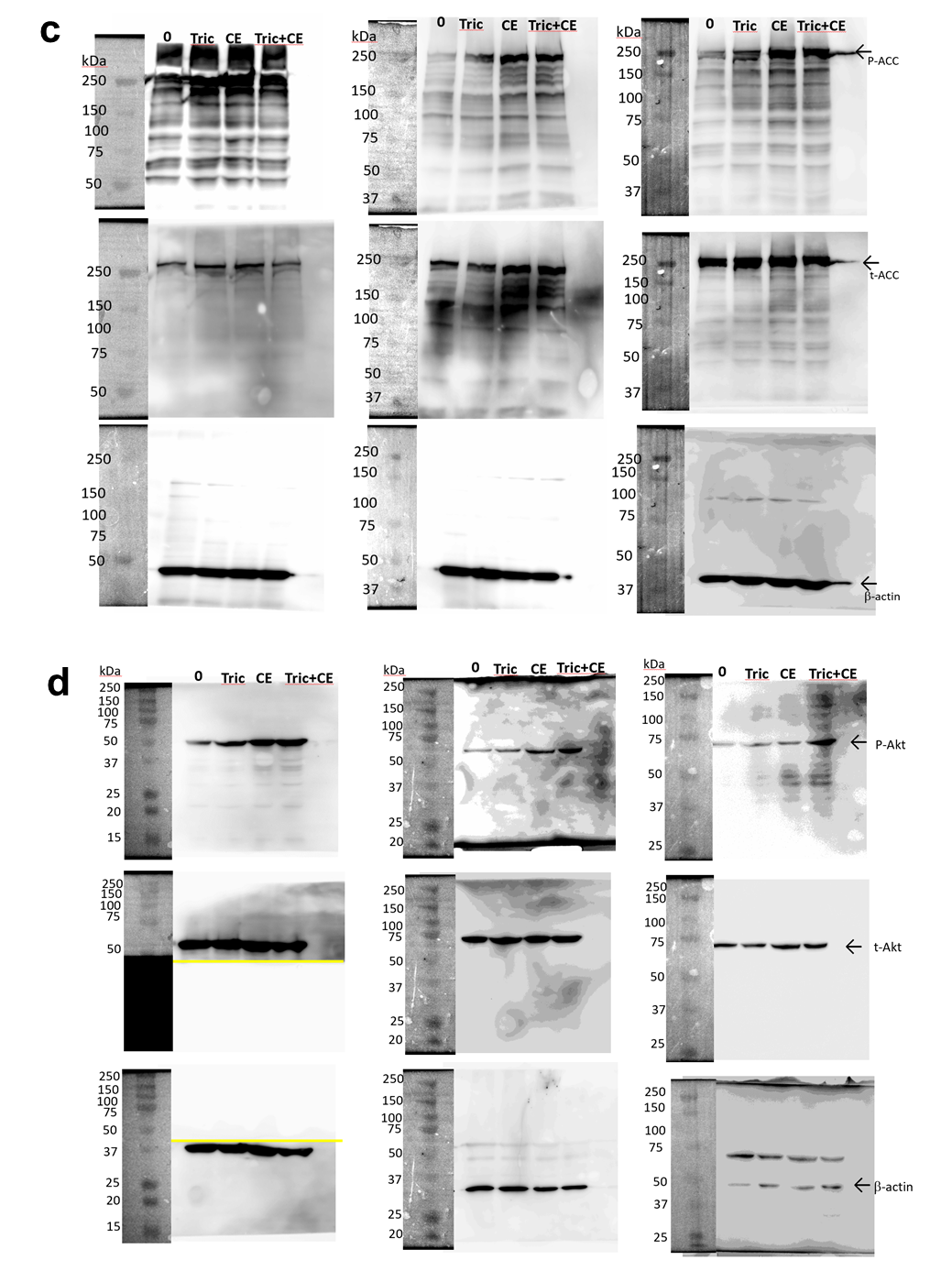

Supplement: Supplementary file 6 — Supplementary Figure S4. [file 41598_2022_13421_MOESM6_ESM.tif]

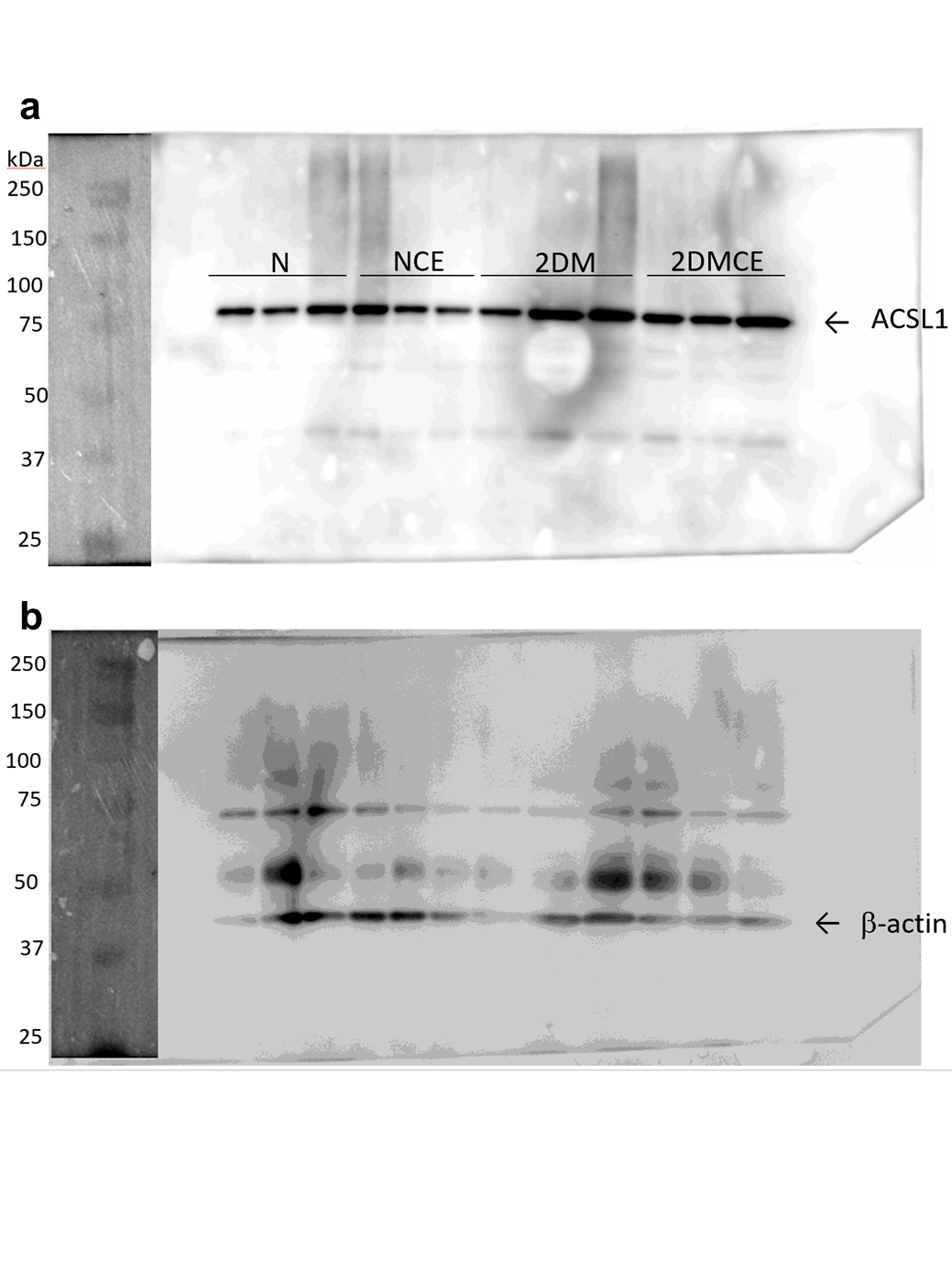

Supplement: Supplementary file 7 — Supplementary Figure S5. [file 41598_2022_13421_MOESM7_ESM.tif]

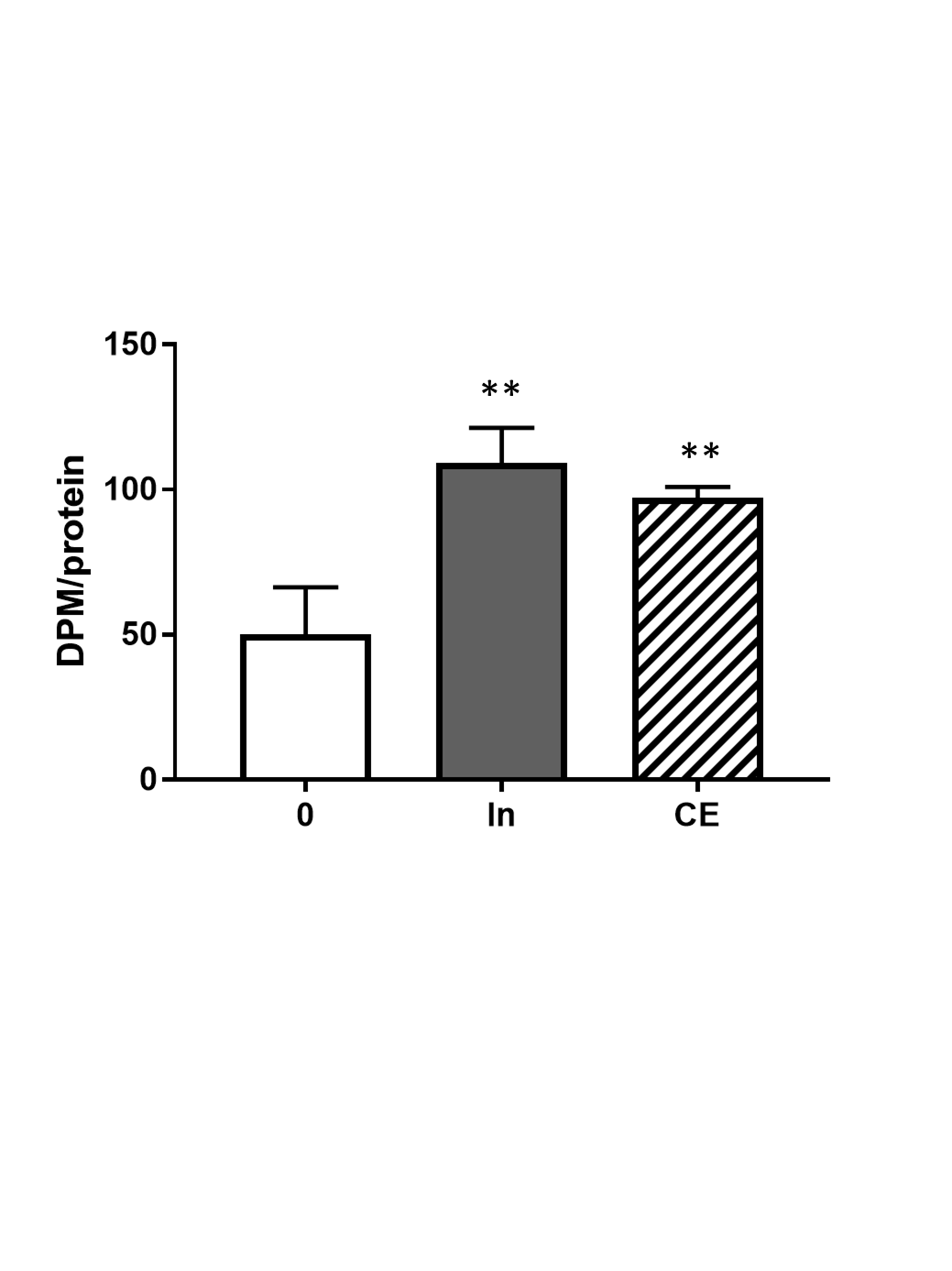

Supplement: Supplementary file 8 — Supplementary Figure S6. [file 41598_2022_13421_MOESM8_ESM.tif]
